# Supplementary material for: Identification of photoperiod-induced specific miRNAs in the adrenal glands of Sunite sheep (Ovis aries)
Source: Front Vet Sci. 2022 Jul 22;9:888207. doi: 10.3389/fvets.2022.888207 (PMC9354845; doi:10.3389/fvets.2022.888207)
Supplement: Supplementary file 1 [file Data_Sheet_1.ZIP › Supplementary file/miRDeep2 file.zip!/miRDeep2 file/Know_Structure/SPLP42A3/oar-mir-3956.pdf]

miRBase precursor : oar-mir-3956
Total read count : 117
oar-miR-3956-5p read count : 113
oar-miR-3956-3p read count : 4
remaining reads : 0

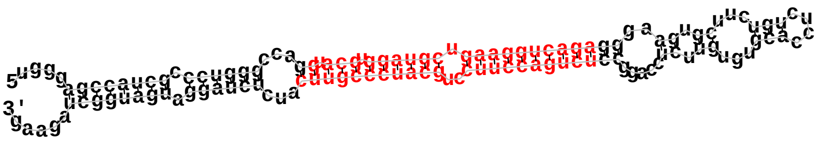

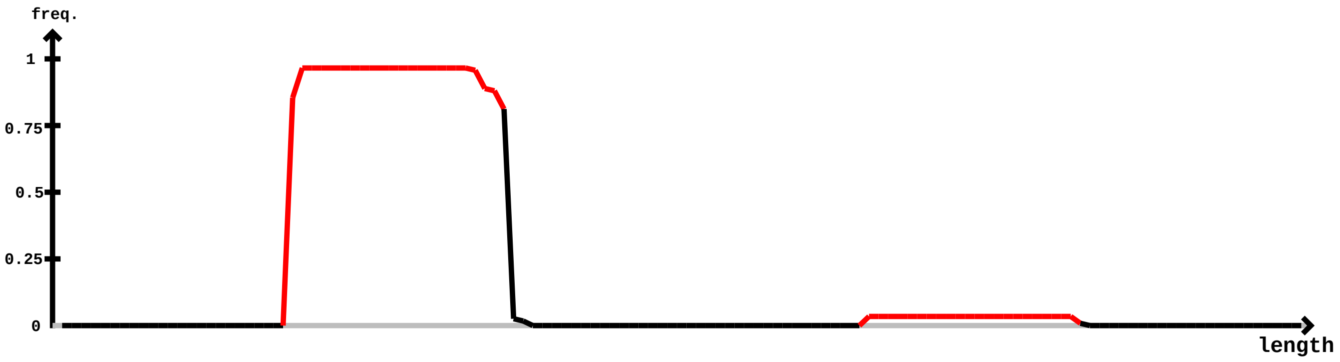


```
                                                                                 oar-miR-3956-3p
                        oar-miR-3956-5p
5'- ugggagccaucgcccugggccagguacguggaugcugaaggucagagggaagugcuucugucuccacguguguucuccaggccucugaccuuccugcaucccguucaucucuaggaugauggcuagaag   -3'    exp
    ....((((((((.((((((...((.(((.((((((.((((((((((((..((.((...(((....)))...)).)).....))))))))))))..)))))))))))...)))))).)))))))).....    reads   mm
    .......................guacguggaugcugaaggu.......................................................................................    1       0
    .......................guacguggaugcugaagguc......................................................................................    7       0
    .......................guacguggaugcugaagguca.....................................................................................    1       0
    .......................guacguggaugcugaaggucag....................................................................................    8       0
    .......................guacguggaugcugaaggucaga...................................................................................    80      0
    .......................guacguggaugcugaaggucagag..................................................................................    1       0
    .......................guacguggaugcugaaggucagagg.................................................................................    2       0
    ........................uacguggaugcugaagguc......................................................................................    1       0
    ........................uacguggaugcugaaggucaga...................................................................................    12      0
    ...................................................................................ucugaccuuccugcaucccguu........................    3       0
    ...................................................................................ucugaccuuccugcaucccguuc.......................    1       0
```
